# Sensorineural Hearing Loss and Its Relationship with Duration of Chelation Among Major β-Thalassemia Patients

**DOI:** 10.7759/cureus.5465

**Published:** 2019-08-22

**Authors:** Muhammad Ali Khan, Mahrukh A Khan, Ahmed M Seedat, Maria Khan, Sana F Khuwaja, Ram Kumar, Syed Muhammad Usama, Sundus Fareed

**Affiliations:** 1 Otolaryngology, Dow University of Health Sciences, Karachi, PAK; 2 Internal Medicine, Dow University of Health Sciences, Karachi, PAK; 3 Family Medicine, Dow University of Health Sciences, Karachi, PAK; 4 Family Medicine, Dow University of Health Sciences, Karachi , PAK; 5 Internal Medicine, Chandka Medical College, Larkana, PAK; 6 Miscellaneous, Dow University of Health Sciences, Karachi, PAK; 7 Internal Medicine, Civil Hospital Karachi, Karachi, PAK

**Keywords:** β–thalassemia, sensorineural hearing loss, chelating agent, pure tone audiogram, chelation complications, iron chelation, pakistan

## Abstract

Introduction

Thalassemia is a common genetic disorder worldwide, also occurring frequently in Karachi, Pakistan. Beta (β)-thalassemia major patients need repeated transfusions which cause iron overload. Patients are treated with chelating agents to reduce the high serum ferritin level and to decrease morbidity and mortality due to increased iron levels. This combined therapy also leads to some complications. One of them is the sensorineural hearing loss (SNHL). To date, no data is available in Pakistan regarding SNHL among major β-thalassemia patients on chelating therapy.

Methods

A cross-sectional study was performed in collaboration with the Thalassemia Center and Dr. Ruth Pfau at the Department of Ear, Nose, and Throat, Civil Hospital, Karachi, Pakistan. The variable to detect hearing was pure tone air and bone conduction thresholds at the frequencies of 250 - 4,000 Hz. Clinical data, such as chelating agent dose, duration, and hearing status, were recorded. Demographic characteristics, like age, gender, height, and weight, were noted. The hemoglobin and serum ferritin levels of the subjects were also included.

Results

Forty-five percent of cases of thalassemia were suffering from SNHL. In the right ear, the Pearson correlation of chelating agent dose (mg) with SNHL was mildly positive and statistically significant (r = 0.261, p < 0.001), (r = 0.337, p < 0.001), (r = 0.198, p = 0.005), and (r = 0.207, p = 0.003) at the frequencies of 250, 500, 1,000, and 2,000 Hz, respectively, and the Pearson correlation of chelating agent used (in months) with SNHL was mildly positive and statistically significant (r = 0.232, p = 0.001), and (r = 0.301, p < 0.001) at frequencies 250 to 500 Hz, respectively. In the left ear, the Pearson correlation of chelating agent dose (mg) with SNHL was mildly positive and statistically significant, (r = 0.191, p = 0.007), (r = 0.202, p = 0.004), (r = 0.297, p < 0.001), (r = 0.183, p = 0.010) and (r = 0.221, p = 0.002) at frequencies 250, 500, 1,000, 2,000, and 4,000 Hz, respectively, and Pearson correlation of chelating agent used (months) with SNHL was mildly positive and statistically significant only at the frequency of 2,000 Hz (r = 140, p = 0.049).

Conclusion

Chelation therapy and regular blood transfusions, apart from prolonging the life of thalassemic patients, also leads to some complications. With this survey, it was concluded that almost half of the patients had normal hearing, while the other half had sensorineural hearing loss after the use of deferasirox. It is inferred that the incidence of SNHL is not only dose-related but the duration of use of a chelating agent is also a contributing factor.

## Introduction

Thalassemia is a hematologic disease first discovered by Thomas Cooley and Lee in 1925 [1]. It is estimated that 5% - 7% of the world's population is affected by a hemoglobin (Hb) disorder [2]. About 3% of carriers are found worldwide with around 60,000 babies being born with genetic mutations resulting in β-thalassemia [3]. It is one of the common disorders in the Mediterranean area [4], the Indian subcontinent, Southeast Asia, West Africa [5], and the Middle East, as well as affecting other countries due to migration [6]. The carrier rate in Pakistan is about 5% - 8%, meaning that up to 9.8 million of the population is affected with around 5,000 babies being diagnosed with the disorder [7]. Consanguinity is a major cause of inheritance of this disease in Pakistan [8]. Thalassemia is a disease characterized by dysfunctional Hb chain formation [5]. It is an autosomal recessive inherited disorder which results in the defective formation of the globin chains manifesting as hypochromic, hemolytic anemia [9]. Thalassemia is among the most common genetic disorders worldwide. Among them, 1.67% of the population is heterozygous and 0.44% of the population is homozygous for alpha and β-thalassemia [10-11]. Alpha-thalassemia is due to absent or reduced synthesis of the alpha-globin chain involving deletion of genes on chromosome 16 [12].

β-thalassemia is a hemoglobinopathy which arises due to a mutation in the Hb subunit β gene on chromosome 11 [10]. It is characterized by the absence or a deficiency in the formation of β globin chains which results in lower Hb levels, resulting in less than efficient transport of oxygen throughout the body; this results in damage to the body due to long-term ischemia and oxygen deprivation of organs and bones, potentially leading to stroke and other complications [13-14]. There are three variants of the disease: β-thalassemia major (a homozygous form which is a severe transfusion-dependent anemia because of the mutation of both of the β globin genes), β-thalassemia intermedia (the mutation is on both globin genes but one is severe and the other one is mild), and β-thalassemia minor (usually asymptomatic and exists as a carrier state with the mutation present in one gene) [10].

The aim of treatment of thalassemia is regular blood transfusions to overcome the reduced blood-making capacity of the body and chelation therapy to decrease the iron load in the body secondary to transfusion and hemolysis. This has helped in adding a number of years to the lives of the diseased. The only definitive cure for thalassemia is bone marrow transplant [15]. The calculation of the serum ferritin level is the most appropriate test to estimate iron overload in β-thalassemia major patients. A desired and safe ferritin level of 1,000 mg/L is generally advocated as a standard practice in thalassemia major and other situations of increased iron from a blood transfusion. When serum ferritin levels approach 1,000 ng/L, this is commonly taken as a stage to begin iron chelation therapy. The main principle of chelation is to maintain a safe iron level threshold to protect the body from developing complications [16].

The introduction of deferoxamine (DFO) (Desferal®, Novartis Pharma Stein AG, Stein, Switzerland) has been a revolution in the field of thalassemia and is the most common iron-chelating agent in use. It reduces cardiac and hepatic disorders and reverses the endocrine dysfunction. However, it can only be administered parenterally and is costly, which reduces its compliance [17]. One study suggested that around 27% of the developed complications are related to hearing on low doses of chelation [18]. A study done by Cohen et al. suggested that there are noticeable side effects on lower doses of DFO but major side effects occur on higher doses, affecting the hearing and resulting in sensorineural hearing loss (SNHL) and induced retinal damage resulting in vision loss later on in life [19].

Similarly, many new oral chelating agents have been introduced in the market, such as deferiprone (DFP) (Ferriprox®, ApoPharma USA, Inc., Rockville, MD) and deferasirox (DFX) (Exjade®, Novartis Pharma Stein AG, Stein, Switzerland), that have relatively fewer side effects than DFO [20]. Adverse side effects of DFP include agranulocytosis which ranges for nine days and is not as severe but has resulted in some deaths and knee arthropathy for which the drug administration was stopped. Gastrointestinal distress, rash, rise in creatinine levels, and liver problems arise with DFX use. Auditory disturbances have been reported when treated with these agents for a long duration of time at high doses and in patients with increased ferritin levels. In most of the cases, the damage was reversible with immediate cessation of the treatment [21]. In our country, no such data is available for SNHL in β-thalassemia patients. It is necessary to study the relationship of SNHL with the duration of use of chelating agents in order to establish newer and better hearing monitoring protocols and improve the quality of life of these patients.

In their 2017 study, Derin et al. highlighted the controversy regarding the ototoxicity of chelating agents [22]. Hence, it is important to identify the high-risk group of patients who are prone to develop SNHL and its relationship with the duration of use.

So far, no data is available regarding SNHL among the β-thalassemia major population in Pakistan. It is important to study the relationship of SNHL with the duration of use of chelating agents, especially when there is a relatively large and adequate population of these patients available, providing insight on how to manage their anemia, along with the complications implicated by the treatment.

The importance of establishing a relationship of SNHL with the duration of use of chelating agents cannot be emphasized enough. Hence, it was imperative to collect such data by conducting this study in order to provide the relationship of SNHL with the duration of use of chelating agents to help to reduce the risk of such complications by adjusting the duration of their treatment and to make changes in their lifestyle and treatment when necessary. This study was aimed to confirm chelating agents induced auditory neurotoxicity and the necessity of periodic audiological control of β-thalassemia patients for prompt diagnosis and management.

## Materials and methods

A cross-sectional study was conducted from January 2014 until December 2017 in collaboration with the Thalassemia Center and the Department of Ear, Nose, and Throat (ENT), Dr. Ruth Pfau, Civil Hospital, Karachi, Pakistan. The study was approved by the Institutional Review Board of Dow University of Health Sciences.

Patients with β-thalassemia major between the ages of five to 25 years who visited clinics for treatment within six months of the start of the study and with no preexisting ear disease were included after informed consent and/or assent from the parents/guardians. Individuals exposed to ototoxic medications other than DFX, cases of congenital hearing loss, acute and chronic otitis media, tympanic membrane perforation found on otoscopic examination, and cases with a history of ear surgeries were all excluded.

A sample size of 200 was calculated using a cross-sectional single population formula with 99.99% confidence interval (CI), prevalence (3.5%), and margin of error (5%). Initially, 208 participants were selected for the study but 10 patients were excluded because of non-compliance and language barriers [23].

Written informed consent was taken, and the procedure and protocols of pure tone audiometry (PTA) (Beltone, Model #120) were thoroughly explained to the patient and/or parents/guardians. Each selected patient underwent ENT examination by the attending otolaryngologist to rule out any current infection, previous surgery, tympanic membrane perforation, or wax in the external auditory canal. PTA was performed free of cost in the Department of ENT; hence, this study did not put any additional monetary burden on the patients and their families. Air conduction was assessed with headphones for the right and left ear separately for frequencies of 250, 500, 1,000, 2,000, 4,000, and 8,000 Hz. Bone conduction was assessed by using the bone stimulator on the mastoid bone of the skull for the right and left ear separately for frequencies 250, 500, 1,000, 2,000, and 4,000 Hz. The readings recorded were plotted on the audiogram chart.

Demographic characteristics, such as age, gender, height, and weight, were noted. The most recent serum hemoglobin (Hb) and ferritin levels were noted. Daily doses of DFX (mg/kg) and duration of use of chelating agent were also recorded.

Data were entered and analyzed on IBM Statistical Package for the Social Sciences (SPSS) Statistics for Windows, Version 21.0. (IBM Corp., Armonk, NY). For quantitative variables, such as age, weight, height, the dose and duration of the chelating agent, Hb, and serum ferritin levels, the mean and standard deviation were calculated. For categorical variables, such as the magnitude of SNHL with frequencies involved compared with the duration and dose of DFX, a Chi-square test was used. The Pearson correlation analysis was used to check the correlation with the chelating agent dose (mg) and duration (months). A p-value of less than 0.05 was considered as being significant.

## Results

There were 198 cases of major β-thalassemia included in this study. There were 126 (63.63%) male and 72 (36.36%) female participants. Their mean age was 135 ± 52 months (range: 60 - 300 months). There were 63 (31.8%) in the age group 60 - 96 months, 75 (37.9%) in age group 97 - 144 months, 42 (21.2%) in age group 145 - 216 months, and 18 (9.1%) participants were older than 216 months. Their mean weight was 1.3 ± 0.2 kg and the mean height was 25.7 ± 9.1 meters.

The mean hemoglobin level of the participants was 7.3 ± 1.5 gm/dL and the mean serum ferritin was 5,555.2 ± 5,431.4 mg/mL. The dose and duration of use of chelating agents are presented in Table 1. Most of the patients (n = 87; 43.9%) were taking 700 to 1,000 mg of the chelating agent per day. A chelation duration of 25 - 60 months was most frequent in the study sample.

**Table 1:** Descriptive Statistics for Dose and Duration of Chelating Agent of Study Participants (n = 198) SD: standard deviation

Variables	Frequency (%)
Dose of chelating agent (mg/day)	
< 700	66 (33.3%)
700 - 1,000	87 (43.9%)
1,000 - 1,800	45 (22.7%)
Mean ± SD	858.3 ± 379.1
Duration chelating agent (months)	
3 - 6	33 (16.7%)
7 - 12	30 (15.2%)
13 - 24	45 (22.7%)
25 - 60	51 (25.7%)
> 60	39 (19.6%)
Mean ± SD	34.5 ± 28.9

The distribution of SNHL in frequencies of 250, 500, 1,000, 2,000 and 4,000 Hz in both right and left ears are presented in Table 2. SNHL was found in 39 (19.69%), 30 (15.15%), and 30 (15.15%) patients at frequencies 500 Hz, 1,000 Hz, and 2,000 Hz, respectively, in the right ear. SNHL was found in 48 (24.2%), 18 (9.1%), and 18 (9.1%) patients at frequencies 2,000 Hz, 500 Hz, and 4,000 Hz, respectively, in the left ear.

**Table 2:** Frequency Distribution of Sensorineural Hearing Loss (SNHL) in Thalassemia Patients (n = 198) dB: decibels; Hz: hertz

Variable	SNHL of Right Ear n (%)	SNHL of Left Ear n (%)
Frequency: 250 Hz		
Normal Hearing: 0 - 25 dB	189 (95. 5%)	189 (95. 5%)
Mild Hearing Loss	9 (4.5%)	9 (4.5%)
Frequency: 500 Hz		
Normal Hearing: 0 - 25 dB	159 (80.3%)	180 (90.9%)
Mild Hearing Loss	39 (19.7%)	18 (9.1%)
Frequency: 1,000 Hz		
Normal Hearing: 0 - 25 dB	168 (84.8%)	183 (92.4%)
Mild Hearing Loss	30 (15.2%)	15 (7.6%)
Frequency: 2,000 Hz		
Normal Hearing: 0 - 25 dB	168 (84.8%)	150 (75.8%)
Mild Hearing Loss	30 (15.2%)	48 (24.2%)
Frequency: 4,000 Hz		
Normal Hearing: 0 - 25 dB	183 (92.4%)	180 (90.9%)
Mild Hearing Loss	15 (7.6%)	18 (9.1%)

**Figure 1:**
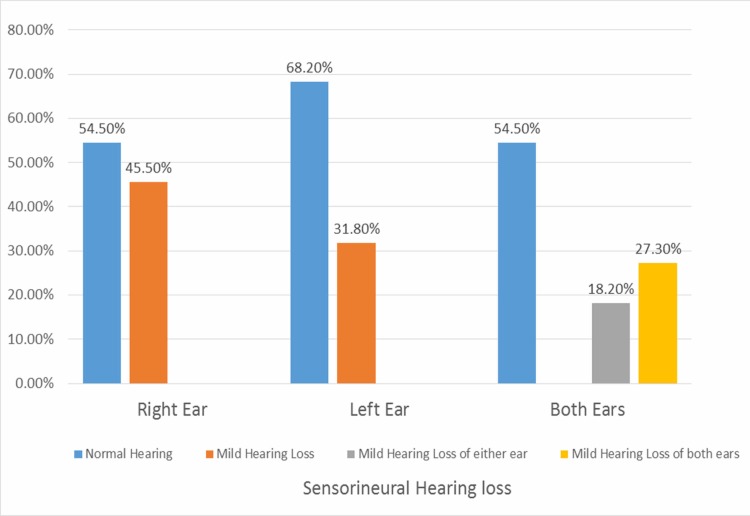
Frequency of sensorineural hearing loss detected in either or both ears in thalassemia patients (n = 198)

In the right ear, 90 (45.5%) patients were detected with SNHL. In the left ear, 63 (31.8%) had SNHL. In both ears, the frequency distribution detected was 36 (18.2%) SNHL in the right or left ear and 54 (27.3%) had SNHL in both ears (Figure 1).

Correlation of the chelating agent dose and duration with the frequency of SNHL of the right ear is presented in Table 3. The Pearson correlation of the chelating agent dose (mg) with SNHL was statistically significant at frequencies of 250, 500, 1,000, and 2,000 Hz (p < 0.001, < 0.001, 0.005, and 0.003, respectively) and that of the duration of chelating agent used (in months) with SNHL was statistically significant (p = 0.232 and 0.301 at frequencies of 250 and 500 Hz, respectively).

**Table 3:** Correlation of the Dose and Duration of the Chelating Agent with the Frequency of Sensorineural Hearing Loss in the Right Ear (n = 198) Hz; hertz; mg: milligrams; p: p-value; r: correlation coefficient; SD: standard deviation

Variable	Pearson correlation	Chelating agent dose (mg)	Duration of chelating agent used (months)	Frequency distribution in the right ear				
				250 Hz	500 Hz	1,000 Hz	2,000 Hz	4,000 Hz
Chelating agent dose (mg)	r	1	.216	.261	.337	.198	.207	.055
	p		.002	<0.001	<0.001	.005	.003	.445
Duration of chelating agent used (months)	r	.216	1	.232	.301	.004	.032	.097
	p	.002		.001	.000	.960	.657	.173
Frequency - 250 Hz	r	.261	.232	1	.893	.635	.556	.291
	p	<0.001	.001		<0.001	<0.001	<0.001	<0.001
Frequency - 500 Hz	r	.337	.301	.893	1	.565	.536	.293
	p	<0.001	<0.001	<0.001		<0.001	<0.001	<0.001
Frequency - 1,000 Hz	r	.198	.004	.635	.565	1	.611	.264
	p	.005	.960	<0.001	<0.001		<0.001	<0.001
Frequency - 2,000 Hz	r	.207	.032	.556	.536	.611	1	.253
	p	.003	.657	<0.001	<0.001	<0.001		<0.001
Frequency - 4,000 Hz	r	.055	.097	.291	.293	.264	.253	1
	p	.445	.173	<0.001	<0.001	<0.001	<0.001	

Correlation of the dose and duration of the chelating agent with a frequency of SNHL of the left ear is presented in Table 4. The Pearson correlation of the chelating agent dose (mg) with SNHL was mildly positive and statistically significant at 0.191, 0.202, 0.279, 0.183, and 0.221 at frequencies of 250, 500, 1,000, 2,000, and 4,000 Hz, respectively. The Pearson correlation of the chelating agent used (in months) with SNHL was weakly positive and statistically insignificant - 0.056, 0.039, 0.140, and 0.056 at frequencies of 250 Hz, 500 Hz, 1,000 Hz, and 4,000 Hz, respectively; however, at the frequency of 2,000 Hz, it was significant at 0.0140 (p = 0.049) (Table 4).

**Table 4:** Correlation of the Dose and Duration of the Chelating Agent and Frequency of the Sensorineural Hearing Loss of the Left Ear (n = 198) Hz: hertz; mg: milligrams; p: p-value; r: correlation coefficient; SD: standard deviation

Variable	Pearson correlation	Chelating agent dose (mg)	Duration of chelating agent used (months)	Frequency distribution in the left ear				
				250 Hz	500 Hz	1000 Hz	2000 Hz	4000 Hz
Chelating agent dose (mg)	r	1	.216	.191	.202	.279	.183	.221
	p		.002	.007	.004	.000	.010	.002
Duration of chelating agent used (months)	r	.216	1	.056	.039	.016	.140	.056
	p	.002		.437	.589	.824	.049	.431
Frequency - 250 Hz	r	.191	.056	1	.918	.729	.580	.440
	p	.007	.437		<0.001	<0.001	<0.001	<0.001
Frequency - 500 Hz	r	.202	.039	.918	1	.769	.516	.384
	p	.004	.589	.000		.000	<0.001	<0.001
Frequency - 1,000 Hz	r	.279	.016	.729	.769	1	.654	.406
	p	<0.001	.824	<0.001	<0.001		<0.001	<0.001
Frequency - 2,000 Hz	r	.183	.140	.580	.516	.654	1	.571
	p	.010	.049	<0.001	<0.001	<0.001		<0.001
Frequency - 4,000 Hz	r	.221	.056	.440	.384	.406	.571	1
	p	.002	.431	<0.001	<0.001	<0.001	<0.001	

Association of the chelating agent dose and duration with SNHL in either the right or left ear and/or both ears is depicted in Table 5. A higher dose and longer duration of chelation were significantly associated with SNHL. The combined association of the dose and duration of the chelating agent with SNHL of the right or left and both ears were statistically significant. In a dosage of 700 - 1,000 mg of the chelating agent, 18 (50%) patients had right or left ear hearing loss, and in the patients using the 1,000 - 1,800 mg dose of the chelating agent, 27 (50%) had hearing loss in both ears. In the duration of > 60 months use of the chelating agent, nine (25%) patients had right or left ear hearing loss and 18 (33.3%) had mild hearing loss in both ears, as depicted in Table 5.

**Table 5:** Association of the Chelating Agent Dose and Duration with SNHL in Either the Right or Left Ear and/or Both Ears (n = 198) HL: hearing loss; SNHL: sensorineural hearing loss

Characteristics of Chelation	Right Ear			Left Ear			Both Ears			
	No HL n (%)	HL n (%)	P-value	No HL n (%)	HL n (%)	P-value	No HL n (%)	HL in Either Ear n (%)	HL in Both Ears n (%)	P-value
Dose (mg/day)										
< 700	39 (59.09%)	27 (40.90%)	<0.001	45 (68.18%)	21 (31.81%)	<0.001	39 (36.11%)	9 (25%)	18 (33.33%)	<0.001
700 - 1,000	60 (68.96%)	27 (31.03%)		72 (82.75%)	15 (17.24%)		60 (55.55%)	18 (50%)	9 (16.66%)	
1,000 - 1,800	9 (20%)	36 (80%)		18 (40%)	27 (60%)		9 (8.33%)	9 (25%)	27 (50%)	
Duration (months)										
3 - 6	15 (45.45%)	18 (54.54%)	0.004	21 (63.63%)	12 (36.36%)	0.007	15 (13.88%)	9 (25%)	9 (16.66%)	0.004
7 - 12	18 (60%)	12 (40%)		24 (80%)	6 (20%)		18 (16.66%)	6 (16.66%)	6 (11.11%)	
13 - 24	30 (66.66%)	15 (33.33%)		36 (80%)	9 (20%)		30 (27.77%)	9 (25%)	6 (11.11%)	
25 - 60	33 (64.70%)	18 (35.29%)		36 (70.58%)	15 (29.41%)		33 (30.55%)	3 (8.33%)	15 (27.77%)	
> 60	12 (30.76%)	27 (69.23%)		18 (46.15%)	21 (53.84%)		12 (11.11%)	9 (25%)	18 (33.33%)	

## Discussion

Of the 198 cases of major β-thalassemia patients, 108 (54.54%) had normal hearing and 98 (45.45%) had sensorineural hearing loss. We found SNHL in β-thalassemia major patients at all doses of DFX from 700 to 1,800 mg (p < 0.001). It was also revealed that SNHL was found in β-thalassemia major patients at all durations of use, from three to 60 months or more (p < 0.007).

This study showed a prominent relationship between the chelating agents and SNHL. Where literature has been reported on the relationship between SNHL and the dosage of chelating agents, not much data exists on the relationship between SNHL and the duration of use of chelating agents in β-thalassemia [18-19, 24]. This study has provided substantial proof regarding hearing loss in β-thalassemia major patients after a longer duration of DFX usage. To the best of our knowledge, this is the first-ever study conducted in Pakistan regarding SNHL in β-thalassemia major patients after the use of DFX and the correlation with its dose and duration of use. However, this study was limited by a low budget, time constraint, lack of cooperation from patients due to the language barrier, and the fact that cases were included from one city only.

A study conducted by Yadav et al. in Haryana, India in 2012 showed that increasing duration of use of the chelating agent caused an increased incidence of SNHL in β-thalassemia major patients [25]. A cross-sectional study carried out by Kong et al. in Malaysia in 2014 showed a 57.4% frequency of SNHL in β-thalassemia major patients. A total of 54 patients were included with the age ranging from three years and above [24]. Another descriptive-analytical study was done by Ashrafi et al. in 2011 on 80 β-thalassemia major patients aged four to 32 years, in which 47% had SNHL with a significant relationship (p < 0.05) of hearing loss with dose and duration of DFX [26].

Kong et al. revealed a significant positive correlation between the dose and frequency of 2,000 Hz and 4,000 Hz on a pure tone audiometry test in the left ear. No significant correlation was observed in the right ear [24]. In another study performed by Osma et al. in 2015 from Turkey, right ear SNHL was 39%, while the left ear SNHL was 27.7% [27]. The findings of these studies are in conjunction with our study.

A study by Vir et al. disclosed that patients taking an injectable iron-chelating agent for three years had a low incidence of SNHL as compared to those taking therapy for six years [28]. Other studies also showed a strong relationship between SNHL with the dose and duration of the chelating agent in β-thalassemia major patients [27, 29].

A negative correlation result between ototoxicity and DFX treatment was reported in a study from Thailand [30]. They analyzed their clinical records from January 1997 to December 2010. All transfusion-dependent thalassemia patients received iron chelation therapy with mono DFX, DFO, or a combination. One hundred thalassemia patients were enrolled and analyzed. SNHL was detected in seven patients but only four were determined to be associated with iron chelators. They reported that there was a rather low incidence of ototoxicity after exposure to iron chelators [30]. In another paper by Derin et al., DFP and DFX were found to be unrelated to SNHL in β-thalassemia major patients [22]. The findings of these studies did not correlate with our study. In our study, 45.45% of patients had SNHL in the right ear compared to the left ear (31.81%). There was a significantly positive correlation between the dose of the chelator and frequencies of 250 Hz, 500 Hz, 1,000 Hz, and 2,000 Hz in the right ear, while a positive correlation between the dose and frequency was observed from 250 Hz to 4,000 Hz in the left ear. Considering the duration, there was a positive correlation at 250 Hz to 500 Hz in the right ear. In the left ear, we found positive correlations at the frequency of 2,000 Hz.

PTA was used to determine the hearing status of patients in this study. In a study conducted by Jiang et al. in 2016, PTA included routine PTA and extended high-frequency audiometry in 60 patients from September 2013 to October 2014 [30]. The results showed that extended high-frequency might be more sensitive in the determination of early SNHL for β-thalassemia major patients. This does not concur with our findings. It is recommended that PTA should be done regularly for every β-thalassemia patient taking a chelating agent. A large study should be done to further evaluate the results. More sophisticated, advanced methods of detection and monitoring the hearing acuity, such as brain-evoked response audiometry and otoacoustic emission, may be performed for a patient who has sensorineural hearing loss.

## Conclusions

Although a definitive cure of thalassemia is a bone marrow transplant, regular blood transfusions (along with chelation therapy) prolong the life of patients. Chelation therapy, while having these benefits, also leads to some complications. With this survey, it was concluded that almost half of the patients had normal hearing, while the other half had sensorineural hearing loss after the use of DFX. It is inferred that the incidence of SNHL is not only dose-related but the duration of use of a chelating agent is also a contributing factor since SNHL was found to be significantly associated with both the dosage and duration of use of a chelating agent.
